# The pros and cons of lung cancer screening

**DOI:** 10.1007/s00330-024-10939-6

**Published:** 2024-07-16

**Authors:** Roberta Eufrasia Ledda, Georg-Christian Funk, Nicola Sverzellati

**Affiliations:** 1 Department of Medicine and Surgery (DiMeC), University of Parma, Parma, Italy; 2 Department of Medicine II with Pneumology, Karl Landsteiner Institute for Lung Research and Pulmonary Oncology, Klinik Ottakring, Vienna, Austria

**Keywords:** Lung cancer screening, Low-dose computed tomography, Benefits and harms

## Abstract

**Abstract:**

Several trials have shown that low-dose computed tomography-based lung cancer screening (LCS) allows a substantial reduction in lung cancer-related mortality, carrying the potential for other clinical benefits. There are, however, some uncertainties to be clarified and several aspects to be implemented to optimize advantages and minimize the potential harms of LCS.

This review summarizes current evidence on LCS, discussing some of the well-established and potential benefits, including lung cancer (LC)-related mortality reduction and opportunity for smoking cessation interventions, as well as the disadvantages of LCS, such as overdiagnosis and overtreatment.

**Clinical relevance statement:**

Different perspectives are provided on LCS based on the updated literature.

**Key Points:**

- *Lung cancer is a leading cancer-related cause of death and screening should reduce associated mortality*.
- *This review summarizes current evidence related to LCS*.
- *Several aspects need to be implemented to optimize benefits and minimize potential drawbacks of LCS*.

## Background

Lung cancer (LC) is the leading cause of cancer-related death worldwide [1]. The premise of lung cancer screening (LCS) is that early detection of LC reduces mortality. Indeed, low-dose computed tomography (LDCT)-based LCS showed a 20–39% reduction of LC mortality in heavy smokers [2, 3]. Based on such evidence, LCS has been increasingly endorsed by national stakeholders and international scientific societies [4–6]. Some uncertainties, however, remain and various aspects need to be implemented to optimize benefits and minimize potential drawbacks of LCS [7].

This review summarizes current evidence on LCS, discussing some of the well-established and potential advantages, including LC-related mortality reduction and opportunity for smoking cessation interventions, as well as the disadvantages of LCS, such as overdiagnosis and overtreatment, with only a minor reduction in all-cause mortality. Potential harms associated with false-positive results, downstream procedures, and management of incidental findings are also of concern.

## Mortality

### Benefits

#### Reduction of lung-cancer-related mortality

The decrease in LC-related mortality by LDCT-based LCS has been well established by randomized controlled trials (RCTs) and meta-analyses [2, 3, 8]. As recently highlighted by Wolf et al [9], several RCTs reported LC mortality results, but only the National Lung Screening Trial (NLST) and the Dutch–Belgian lung-cancer screening (NELSON) trial were adequately powered to assess the association between LC mortality and an invitation to screening [10]. Namely, the NLST demonstrated a 20% relative reduction in LC mortality at a median of 6.5-years follow‐up [2], while results from the NELSON trial showed an overall 25% relative reduction in LC deaths, with a 24% relative reduction among men and a 33% among women [3]. These more favorable effects of LCS on LC-related mortality among women participants were also demonstrated by the German LCS trial (LUSI) [11] and by the multicentric Italian lung detection (MILD) and BioMILD trials [12].

### Harms

#### Minor reduction in all-cause mortality

Overdiagnosis and overtreatment within a patient population result in decreased disease-related mortality without a corresponding reduction in all-cause mortality. This outcome arises because screening leads to the detection and successful treatment of more cases, thus lowering the number of deaths attributed to that given disease. However, no or only a minor reduction in all-cause mortality is observed. This is often because the disease detected and treated was either not severe enough to significantly impact all-cause mortality or because the life expectancy of the population was already compromised by other substantial comorbidities, leading to deaths from different causes. This phenomenon is exemplified by prostate cancer screening, whereby the increased detection and treatment of prostate cancer leads to a decrease in deaths from prostate cancer but does not affect all-cause mortality rates. The detected cases of prostate cancer often do not pose a high mortality risk, and patients may die from other causes [13, 14].

In the context of LCS, meta-analyses have thus consistently shown a substantial relative reduction in LC-specific mortality [8, 15–18]. Three out of four meta-analyses observed no significant all-cause mortality reduction, with one showing only a slight reduction [8, 16–18]. A recent systematic Cochrane review showed a slight relative decrease in all-cause mortality of about 5% [15].

One large real-world study from China described both a reduction in LC-specific mortality and all-cause mortality, but the effect sizes were minute: the number of subjects who need to be screened to prevent one death due to LC was roughly 1000, and the number of those who would require screening to prevent one death of any cause was approximately 500 [19].

Summary statement: LDCT-based LCS has been proven to reduce LC-related mortality, but a substantial reduction in overall mortality is still to be demonstrated.

## Diagnosis and treatment

### Benefits

#### Stage shift towards early-stage lung cancer

Analogously to any screening, LCS favors the shift of the LC stage from advanced forms (stage III–IV) toward early curable disease, with positive implications in terms of survival. Notably, several trials demonstrated that LDCT-based LCS was effective in reducing LC-related mortality due to a “stage shift” toward early-stage disease with 60–70% of LCs detected at stage I [2, 3, 20].

#### Conservative approach for less aggressive lung cancer

**Fig. 1:**
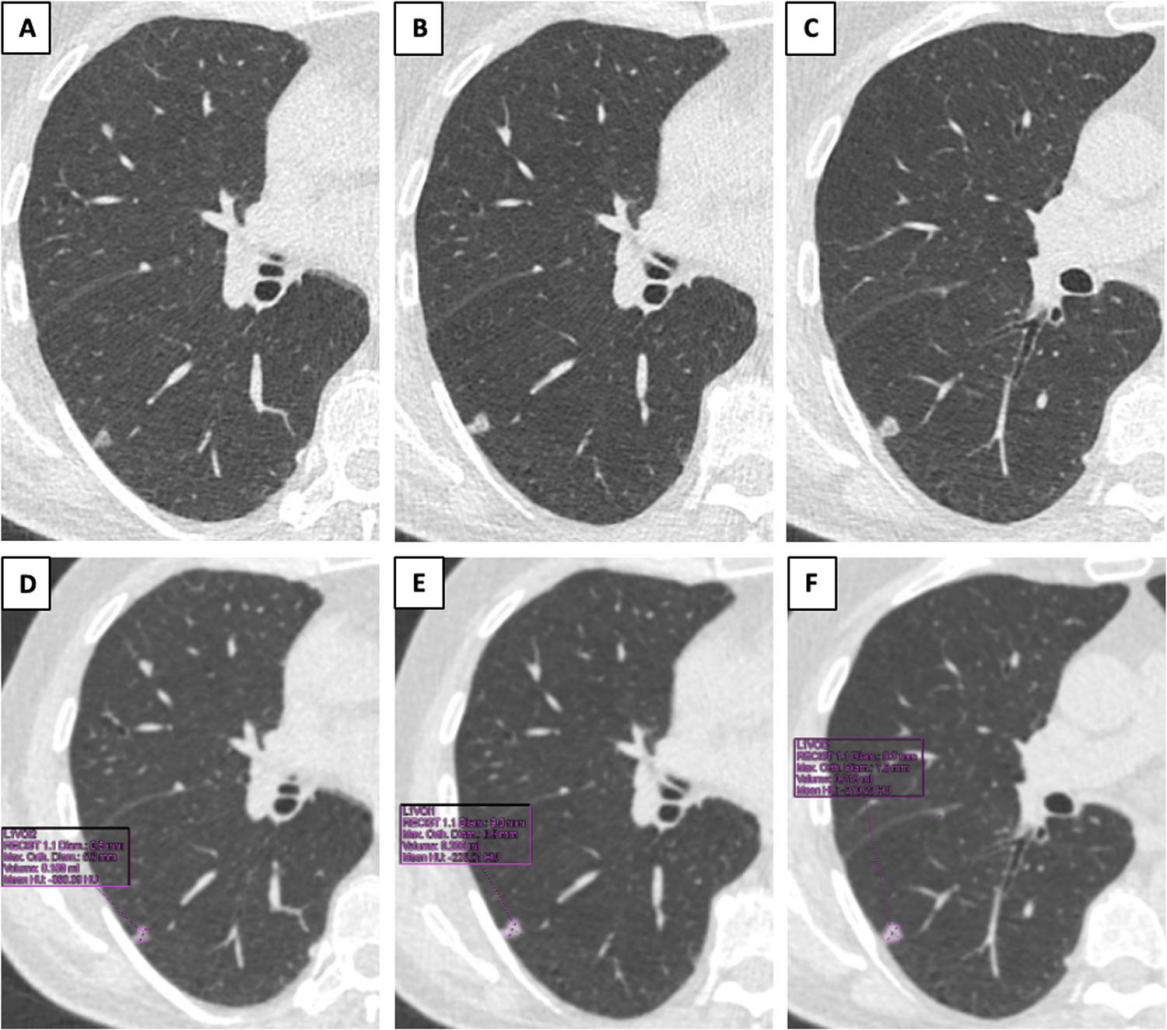
Example of a lung adenocarcinoma presenting as a non-solid nodule at baseline LCS low dose computed tomography (**A**), which developed a solid component at 12-months follow-up (**B**). A further increase in nodule size and density is shown in **C**. Nodule size and density for the three-time points are shown in **D**–**F**, respectively

Any screening is supposed to carry the risk of overdiagnosis and overtreatment, whereby cancers that would have not affected life expectancy because of their less aggressive behavior and/or participant’s comorbidities undergo invasive treatment [21]. It has been shown that less aggressive LCs more commonly manifest as subsolid nodules (Fig. 1), reflecting a preinvasive or minimally invasive histology [22–24]. Although subsolid nodules have a higher likelihood of malignancy as compared to solid nodules, they tend to have indolent clinical behavior and long-term survival without intervention [25]. The progression toward more invasive forms is demonstrated by the development of a solid component or by an increase in the size of a pre-existing solid component. As observed by Silva et al [26], these morphological changes, typically occurring at a slow rate, can be safely detected on serial LDCT performed within an LCS setting. Based on current evidence, long-term LCS may offer an opportunity for a more conservative approach to screening detected subsolid nodules.

### Harms

#### Overdiagnosis and overtreatment

**Fig. 2:**
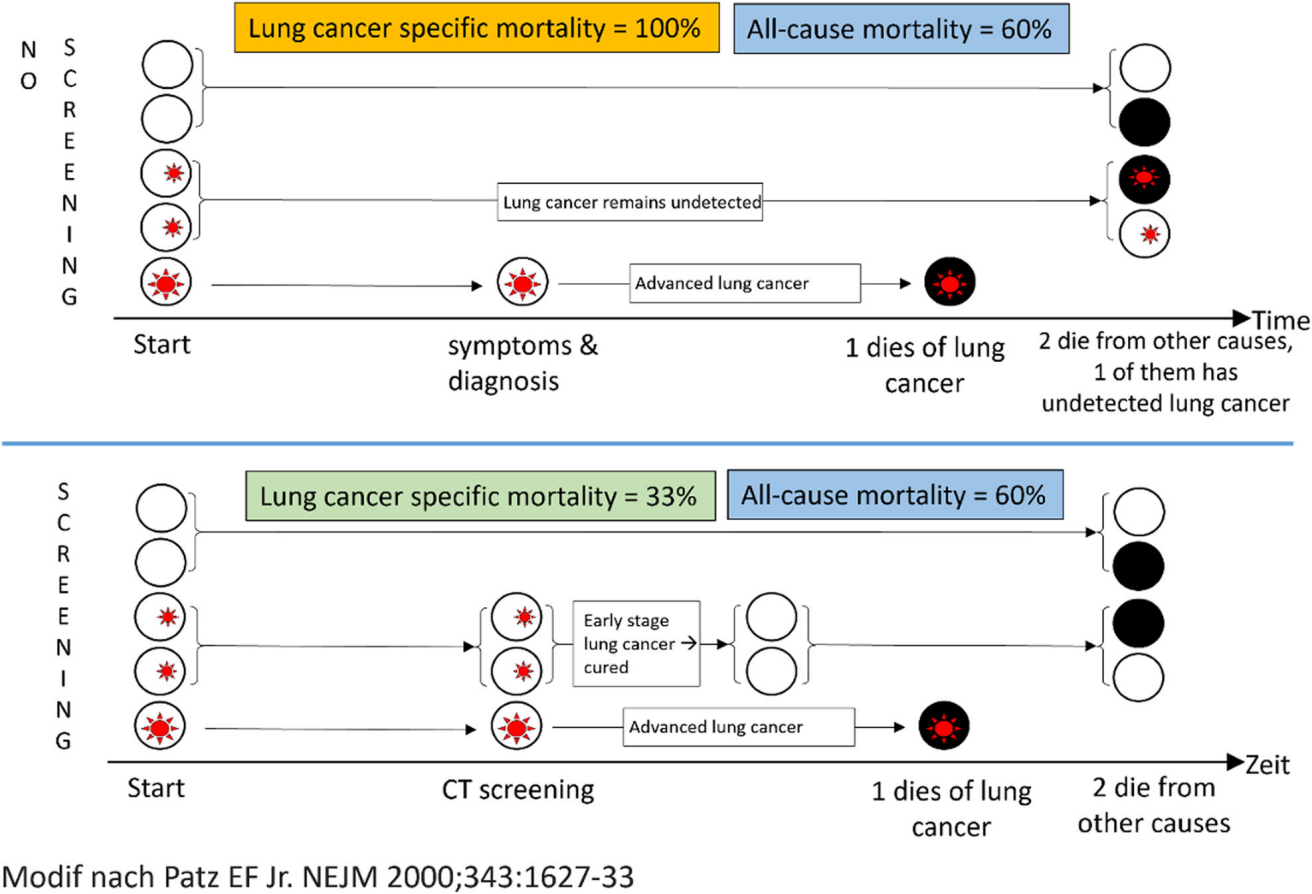
Graphic representation of the effect of LCS on lung cancer-specific and all-cause mortalities

Overdiagnosis means turning people into patients unnecessarily by identifying medical problems that would have never caused them harm because they would have not become clinically apparent in their lifetime [27]. The classic example is a 90-year-old frail and multimorbid man with newly diagnosed prostate cancer who will unlikely live long enough for the cancer to cause him harm. In the context of LCS, this relates to slow-growing adenocarcinomas [28]. Even if such a cancer is detected early and cured, the patient might die later from a competing cause of death, such as cardiovascular (CV) diseases [21, 29]. The typical consequence of overdiagnosis is overtreatment: an intervention that does not benefit the patient or where the risk of harm from the intervention is likely to outweigh any benefit the patient will receive [30, 31]. In the context of LCS, overtreatment happens when a patient with reduced life expectancy due to substantial comorbidity is screened, diagnosed with non-aggressive LC, and treated with surgery or curative radiation [20, 32–35]. The fact that this problem applies to LCS was recognized almost a quarter of a century ago, as shown in Fig. 2 [29].

Calculating the rate of overdiagnosis in screenings is difficult, as the estimation of excess cancers is affected by the length of follow-up periods [36, 37]. It is estimated that almost one in five lung cancers identified through LCS are overdiagnosed [15, 38, 39]. A Cochrane review estimated that seven cases of LC overdiagnosis would occur for every 1000 people screened (95% confidence interval of 2–84 instances of overdiagnosis) [15].

#### False positive results and downstream procedures

False positive results are abnormalities that turn out not to be diseases after further investigation [27]. The rate of false positives showed significant variation across different studies, likely due to inconsistent radiological definitions of positive results. A review conducted for the U.S. Preventive Services Task Force revealed that false-positive rates ranged from 8% to 49% for baseline screening rounds and from 1% to 29% for subsequent rounds [10]. Three studies investigating the impact of the use of Lung-RADS on false positive rates, observed that even in the case of Lung-RADS use the rates of false positives remained between 10% and 25% [40–42]. An analysis based on NLST data, however, suggested that the implementation of Lung-RADS criteria could prevent approximately one out of four invasive procedures due to false positive results [42].

A real-world analysis conducted through the Veterans Administration demonstrated even higher rates of false-positive results, namely 29% among veterans eligible for LCS and 58% among those who were enrolled (at the baseline round) [43].

In the NLST, 17 in 1000 subjects with false-positive results underwent an invasive diagnostic procedure (needle biopsy, thoracotomy, thoracoscopy, mediastinoscopy, and bronchoscopy), and 0.4 in 1000 suffered from significant complications [33]. A systematic Cochrane review reported that invasive tests were higher in the screened group, in whom, however, the risk of post-surgical mortality was not increased [15]. A real-world LCS study showed substantially higher absolute rates of downstream imaging and invasive procedures in screened patients compared to the NLST: 32% and 3%, respectively. In patients undergoing invasive procedures after abnormal findings, complication rates were substantially higher than those in NLST (31% vs 18% for any complication; 21% vs 9% for major complications) [44].

#### Psychological harms

It has been demonstrated that subjects who received an indeterminate result after LCS may have short-term increased distress levels [45]. On the other hand, a systematic review has shown that the CT screening group felt less anxious as compared to the control group who were not offered to participate in LCS [15]. In summary, LCS does not seem to have net adverse psychological effects.

#### Radiation exposure

Several parameters should be considered to evaluate the risk associated with radiation exposure in LCS. These include screening-related factors, such as CT protocols, screening interval and duration, and participant-related factors like age at the enrollment, gender, and tobacco exposure. It is estimated that LCS-related radiation exposure is currently around 1.5 mSv per year, with higher levels to be considered in the case of additional diagnostic CT scans for indeterminate and positive results [5]. Repetitive scans would result in one radiation-related cancer death every 2500 screenees [46], posing the need for radiation dose optimization.

According to the updated literature, the radiation burden from LDCT is probably a minor issue due to currently available ultra-low dose CT protocols [47, 48].

Summary statement: there is growing evidence suggesting that the benefits of LCS go beyond the reduction of LC-related mortality, with the possibility of more conservative approaches for screen-detected LCs. There are, however, several aspects to be implemented to minimize potential drawbacks.

## Incidental findings

### Benefits

#### Detection of pulmonary findings with potential clinical significance

**Fig. 3:**
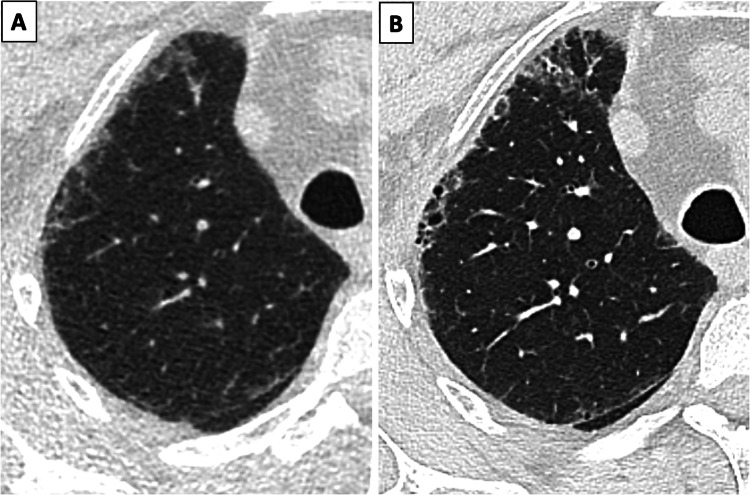
Example of ILA detected on baseline LCS low dose computed tomography (**A**), with evidence of progression toward over fibrosis at standard dose chest CT (**B**) performed after 36 months

Pulmonary incidental findings are extremely common on LDCT-based LCS, being detected in up to 70% of subjects [49]. Among them, emphysema, and interstitial lung abnormalities (ILA) (Fig. 3) are the most frequently reported.

Regardless of its type and extent, emphysema is reported in 24–63% of LCS participants, in whom the presence of emphysema has been proven to be independently associated with higher rates of LC incidence and mortality as well with increased risk of all-cause and respiratory disease-related mortality [50, 51]. Therefore, the importance of detecting and reporting emphysema in LCS is not so much related to early intervention, which should include referral for clinical and functional assessment of those with moderate-severe (> 25% of lungs involved) emphysema [52], but to the concept of a risk-based approach, whereby higher-risk subjects should be offered shorter screening intervals and long-term screening duration. Aiming at reducing the high intra- and interobserver variability of visual assessment and at improving the work-loads in LCS, several studies tested the performance of a quantitative approach (e.g., based on the percentage of low attenuation areas, %LAA) for both detection and quantification of emphysema in screening cohorts [53–55], demonstrating the feasibility of such approaches. Durawa et al, however, reported that visual assessment of emphysema seems to be superior to automated evaluation in terms of prediction of LC risk in LCS participants [56]. Indeed, the visual quantification of emphysema as mild (< 25%), moderate (25–50%) or severe (> 50%) is highly recommended by the ERS/ESTS/ESTRO/ESR/ESTI/EFOMP statement on management of incidental findings in LDCT-based LCS [52].

ILA is defined as subclinical interstitial changes incidentally detected in subjects undergoing either abdominal or chest CT examination, without any clinical suspicion of underlying interstitial lung disease [57]. The estimated prevalence of ILA in LCS populations ranges between 4% and 20% [58]. The relationship between ILA and LC has been investigated in some LCS trials, and a higher prevalence of LC in screenees with ILA has been established [59], highlighting the importance of a risk-based LCS. Nevertheless, the clinical significance of ILA in the setting of LCS goes beyond the increased risk of LC. Indeed, some morphological subtypes of ILA, namely subpleural fibrotic, are associated with a higher risk of progression toward overt pulmonary fibrosis [60]. Clear guidelines on how ILA should be managed in the setting of LCS are still awaited. Quantification and characterization in non-subpleural, subpleural non-fibrotic and subpleural fibrotic are recommended, but whether the detection of ILA should prompt early referral for respiratory evaluation or managed with surveillance imaging is still to be assessed [52].

Given the LCS eligibility criteria, especially in Western countries, overt pulmonary fibrosis, as well as non-fibrotic smoking-related abnormalities (i.e., respiratory bronchiolitis, RB) can also be detected on LCS LDCT [49]. Whether such abnormalities should be considered “incidental” in heavy smokers is debated.

#### Improvement of CV risk assessment

LDCT-based LCS allows the detection and quantification of thoracic findings beyond lung parenchyma, including coronary artery calcification (CAC), which is an independent predictor of CV events and mortality [61]. Being LCS participants mostly heavy smokers, coronary calcium is frequently noted on LCS CT scans [62, 63]. Despite initial skepticism around the possibility for CAC quantification on non-ECG gated scans, recent evidence demonstrated the feasibility of CAC measurement on chest LDCT [64]. CAC is commonly scored by the Agatston score [65], which can be assigned either visually or through artificial intelligence (AI)-based automated approaches [54]. There have been numerous studies that investigated the relationship between CAC and LCS across US and Europe, as well as in Asian countries. Results from both the NLST and the Early Lung Cancer Action Project showed a positive correlation between CAC and CV-related mortality [66, 67], while higher CAC scores, namely > 400, were found to be significantly associated with also all-cause mortality in some European trials, including the NELSON, the Danish Lung Cancer Screening trial (DLCST) and the MILD trial [68–70]. Based on this evidence, the ROBINSCA trial has been investigating whether coronary calcium-based management decreases CV-related mortality and morbidity, with preliminary results showing that CAC score classified significantly less high-risk LCS participants as compared to the clinical Systematic Coronary Risk Evaluation (SCORE) [71].

#### Body composition analysis

There is evidence linking body composition with clinical and prognostic outcomes in LC patients [72]. Several metrics and methods have been proposed to assess body composition, including body mass index and analysis of a single cross-sectional image extracted from either CT or magnetic resonance imaging (MRI) at a certain level (e.g., T12, L1, or L2 vertebral bodies) [73–75]. More recent approaches are based on the automated evaluation of the whole CT or MRI volume data [76]. Although body composition analysis is still very limited in the setting of LCS, it seems to have a potential for targeting higher-risk participants. Indeed, Xu et al have recently observed that an AI-based fully automated measurement of body composition, derived from baseline LDCT evaluation, added predictive value for all-cause mortality, LC-related and CVD-related mortality in the NLST [77].

### Harms

Research has shown a significant variation in the rate of incidental findings among different LCS trials, ranging from 4% to 41% [43, 78–84]. This wide range is attributable to the absence of a uniform definition for what represents an incidental finding. Typical incidental findings encompass both thoracic abnormalities, such as CAC, aortic aneurysms, emphysema, ILA, and signs of pulmonary infection [49], as well as extra-thoracic ones, including kidney, breast, adrenal gland, liver, thyroid, pancreas, spine, lymph node masses, nodules, or cysts. These incidental findings often require further diagnostic work-up, such as consultations, additional radiological exams, and even invasive procedures, which come with their economic costs and potential clinical complications. The risk of overdiagnosis and overtreatment in the setting of LCS does not apply only to non-aggressive LCs, but also to incidental findings. The true benefit of incidental findings lies in their potential to prompt beneficial lifestyle changes or medical treatment with a demonstrable positive impact on relevant health outcomes such as overall mortality and quality-adjusted life years. However, the effectiveness of reporting incidental findings on these potential outcomes remains uncertain in LCS.

Summary statement: although chest LDCT allows the detection of abnormalities besides pulmonary nodules, further research is needed to assess the effectiveness of reporting such findings. Also, a clear definition of incidental findings in the setting of LCS is still to be provided.

### Smoking cessation

Smoking cessation deserves a separate discussion as no real harm can be identified relating to such a topic, which is considered the most important behavior change to reduce LC-specific mortality in LCS cohorts [85–87]. LCS attendance may provide an excellent opportunity for smoking cessation intervention as participants are likely to be more concerned with their health as compared to the eligible non-screening participants. Indeed, some research suggests that LCS represents a “teachable moment” for smoking cessation, whereby current smokers screenees might be particularly receptive to offers of assistance to quit smoking [88]. Various studies, including RCTs, have demonstrated that integrated—rather than delivered within usual care services—and more intensive smoking cessation interventions (i.e., multiple counseling classes with or without pharmacological therapies) are more effective in motivating screening participants to quit smoking as compared to less intensive interventions. Recently published results from the Korean Lung Cancer Screening Project (K-LUCAS) showed that 24.3% of participants had stopped smoking after 6 months from the initial screening [89]. Similarly, the DLCST reported smoking cessation rates of 10.9% at 1-year follow-up [32], the UK Lung Cancer Screening Trial (UKLS) and the German LUSI trial reported rates of 23.6% and 12.8% at 2 years, respectively [90, 91], while the Italian Lung Study (ITALUNG) a rate of 20, 8% at 4 years after baseline [92]. Based on such evidence, recent guidelines released across Europe and the USA strongly recommend incorporating smoking cessation interventions into LCS programs [4, 5, 9]. Nevertheless, there is still little guidance on how such interventions ought to be delivered and limited evidence exists on the effectiveness of different approaches, with only a few trials testing different smoking cessation strategies [92–94].

## Conclusion

LCS has been proven to reduce LC-related mortality, with still controversial evidence on the effect on all-cause mortality reduction. LCS can offer an opportunity for CV diseases and respiratory diseases secondary prevention but carries the risk of false-positive results leading to unnecessary tests and invasive procedures for both non-aggressive lung cancer and incidental findings. Further research into personalized screening intervals and risk prediction models is needed to enhance the benefit-to-harm ratio.

## Abbreviations

CAC: Coronary artery calcification
CV: Cardiovascular
ILA: Interstitial lung abnormalities
LC: Lung cancer
LCS: Lung cancer screening
LDCT: Low-dose computed tomography
MILD: Multicentric Italian lung detection
MRI: Magnetic resonance imaging
NLST: National Lung Screening Trial
RCTs: Randomized controlled trials
